# ﻿*Wedeliafigueiredoana* (Asteraceae, Heliantheae), a winged cypselae new species of *Wedelia* for Brazil

**DOI:** 10.3897/phytokeys.249.135699

**Published:** 2024-11-12

**Authors:** Vinicius R. Bueno, Leonardo S. Rodrigues, Francisco Diego Sousa, Izaías C. Souza, Juliana Marzinek, Danilo Marques

**Affiliations:** 1 Fundação de Parques Municipais e Zoobotânica, Herbário BHZB, Belo Horizonte, MG 31365-450, Brazil Fundação de Parques e Zoobotânica Belo Horizonte Brazil; 2 Universidade Estadual Vale do Acaraú, 62040-370, Sobral, Ceará, Brazil Universidade Federal de Uberlândia Uberlândia Brazil; 3 Universidade Federal do Ceará, Programa de Pós-Graduação em Ecologia e Recursos Naturais, Campus do Pici, Centro de Ciências, Bloco 902, 60440-900, Fortaleza, Ceará, Brazil Universidade Estadual Vale do Acaraú Sobral Brazil; 4 Universidade Estadual Paulista, Programa de Pós-Graduação em Agronomia, 15385-000, Ilha Solteira, São Paulo, Brazil Universidade Federal do Ceará Fortaleza Brazil; 5 Universidade Federal de Uberlândia, Instituto de Biologia, Campus Umuarama, Bloco 2D, 38.400–902 Uberlândia, Minas Gerais, Brazil Universidade Estadual Paulista Ilha Solteira Brazil

**Keywords:** Auricular projections, Cerrado, Compositae, fruit anatomy

## Abstract

We describe one new species from Ceará state, Brazil: *Wedeliafigueiredoana*. It is morphologically associated with *W.bonplandiana*, but distinguished by 0.25–1.05 cm leaf blade width (vs. 1.5–2.6 cm), linear to narrow oblong leaf blade (vs. elliptic to spatulate), 3-seriate involucre (vs. 2-seriate), and cypselae 3.9–4 mm long (vs. 6–7 mm). An anatomical analysis of cypselae is provided, and we propose a more accurate terminology to describe similar wings to *Wedelia* species. We also provide a scientific illustration of the new species, photos of habitat, a map of its geographic occurrence, and its taxonomic affinities are discussed with a taxonomic key to the *Wedelia* species with the apically pronounced wings in the cypselae.

## ﻿Introduction

*Wedelia* Jacq. is inserted in Heliantheae Cassini. This genus occurs mainly in the Neotropics (Strother 1991; Panero 2007), and occasionally in the south USA and Tropical Africa (Strother 1991). We adopted the *Wedelia* circumscription of Turner (1992), which includes *Aspilia* Thouars as its synonym. Brazil is the most rich-species country of the world for the genus with the occurrence of 89 species, 56 of them endemic (Flora e Funga do Brasil 2024).

*Wedelia* can be characterized by herbaceous or shrubby habit, opposite leaves, blades lanceolate to ovate, oval or elliptic; capitula solitaires or in cymes, radiate, rarely discoid; 2–4-seriate involucre, paleaceous receptacle; pistillate or sterile ray florets, yellow or yellow to orange corolla; bisexual disc florets, yellow to orange corolla; black anthers, yellow styles arms; black cypselae, winged or no-winged, coroniform pappus, 0–3 awns (Panero 2007).

During field expeditions in the municipality of Graça, Ceará state, Brazil in February 2023, specimens with yellow florets of Asteraceae were found. After extensive literature review, morphological analysis of specimens from herbaria, and anatomical studies led to the conclusion that this is a new species with unusual cypselae structures, when compared with the Brazilian *Wedelia* species. Thus, we propose here a new species of *Wedelia* for the Brazilian flora. We provide morphological description, an illustration plate, photos and information on the habitat, distribution map, preliminary data on its conservation status, taxonomic key to the Brazilian *Wedelia* species with winged cypselae, and the morphological relationship with the most similar species is discussed. In addition, an anatomical plate is provided and the taxonomic implications for *Wedelia* of its results are discussed.

## ﻿Material and methods

Herbarium specimens from BHCB, BHZB, CEN, HUFU, ICN, R, RB, SPF (acronyms according to Thiers 2024) were analyzed. Several online herbarium databases were consulted, including the following: C. V. Starr Herbarium Virtual (https://sweetgum.nybg.org/science/vh/), Reflora (http://reflora.jbrj.gov.br/reflora/), Smithsonian Virtual Herbarium (https://collections.si.edu/search/), SpeciesLink (https://specieslink.net/), and Tropicos (https://tropicos.org). The types of numerous *Wedelia* species were consulted for taxonomic comparisons. The literature of *Wedelia* was reviewed (Baker 1884; Turner 1988, 1992, 2004; Strother 1991; Pruski and Robinson 2018; Bueno et al. 2019; Bueno and Nakajima 2020; Remor et al. 2022; Flora e Funga do Brasil 2024).

The morphological description was based on vegetative and reproductive material from herbaria specimens, for which a stereomicroscope was used to perform the measurements with a caliper rule. The vegetative structures were described from dried material, whereas reproductive structures were characterized after rehydration, immersing the structure in warm water for 1 minute at 100 °C. The outliers of measures were based on calculation of medians, quartiles, and interquartile deviations. “Rarely” is applied for characters that occur in up to 10% of the specimens studied; “sometimes” is adopted for features that occur in between 10.01% and 25% of the specimens analyzed; “often” is used for the characters that occur between 25.01% and 40% of the specimens studied; and “Or” is applied for traits that occur between 40% and 60% of the specimens (Bueno and Heiden 2022).

The general morphological terminology follows Hickey (1973), Ellis et al. (2009), and Beentje (2016). The specialized Compositae terminology follows Funk et al. (2009), and specific literature about the terminology of receptacle (Bueno et al. 2022) and pappus (Bueno and Heiden 2022). Although Bueno and Heiden (2022) proposed the terminology of pappus based on pappus scales, they refer to the size of pappus scales and the frequency of different lengths in the same heads. We believe that this terminology contributes to the description of the aristate pappus of *Wedelia*. The GeoCAT analysis (Bachman et al. 2011) and IUCN guidelines (2022) were used for preliminary conservation status assessment. The distribution maps were prepared in QGIS v. 3.0 (QGIS Development Team 2015).

We used a technique for scanning electron microscopy (SEM) to observe the details of the surface of the cypsela. Mature ray and disk cypsela were placed on aluminum stubs and then covered by gold old using a sputter coater (Leica EM SCD050). Pictures of the cypsela were taken from a SEM (Tescan VEGA 3 LMU). Anatomical studies were made from exsiccatae, therefore, the cypselae were rehydrated in a solution containing 5% NaOH for four hours (Anderson 1963 modified). After that time, the material was washed with distilled water for 24 hours, and dehydrated in an increasing ethylic series. Dehydrated cypsela was included in historesin (Leica®) following the manufacturer protocol. The material was 8–10 μm thick on a rotary microtome and the sections obtained were stained in toluidine blue at pH 4.7 with acetate buffer (O’Brien et al. 1964 modified). Finally, the sections were mounted in synthetic resin and images were taken using a light microscope (Olympus BX51). The anatomical terminology pericarp follows Roth (1977).

## ﻿Results

### ﻿Taxonomy

#### ﻿ Wedelia figueiredoana

Taxon classificationPlantaeAsteralesAsteraceae

V.R.Bueno sp. nov.

2B37D960-1F84-5634-90D9-C42EFF389307

urn:lsid:ipni.org:names:77351701-1

**Figure 1.**
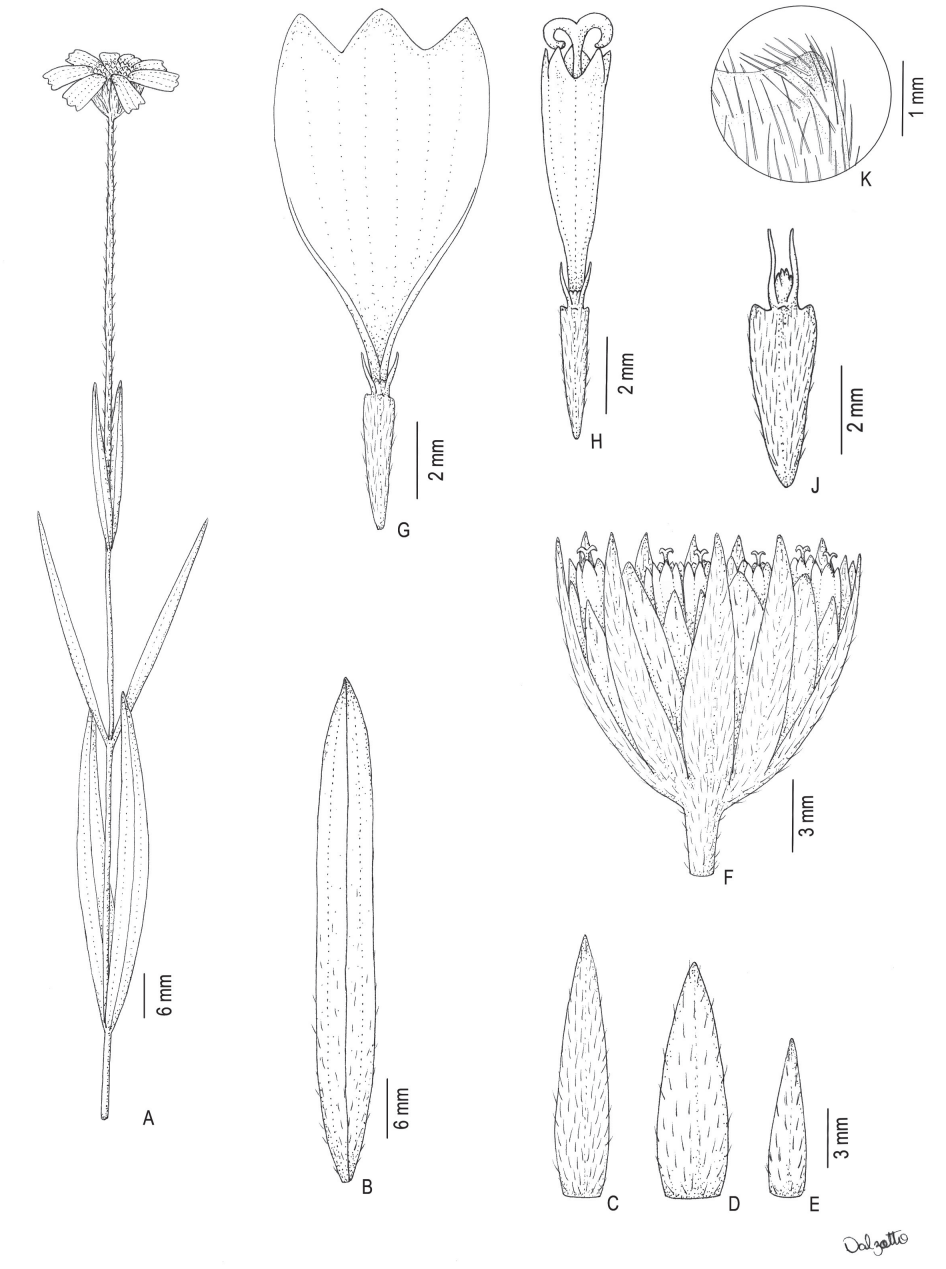
*Wedeliafigueiredoana* sp. nov. **A** flowering branch **B** abaxial surface leaf **C** first series of phyllaries **D** second series of phyllaries **E** third series of phyllaries **F** capitulum with ray florets removed to show involucre, paleae, and disc floret arrangements **G** ray floret **H** disc floret **J** cypselae with apically pronounced wings **K** apically pronounced wing **A–K** drawn from L. S. Rodrigues 201 (HUFU) **A–K** millimeter scale. Illustration by Débora Dalzotto.

**Figure 2.**
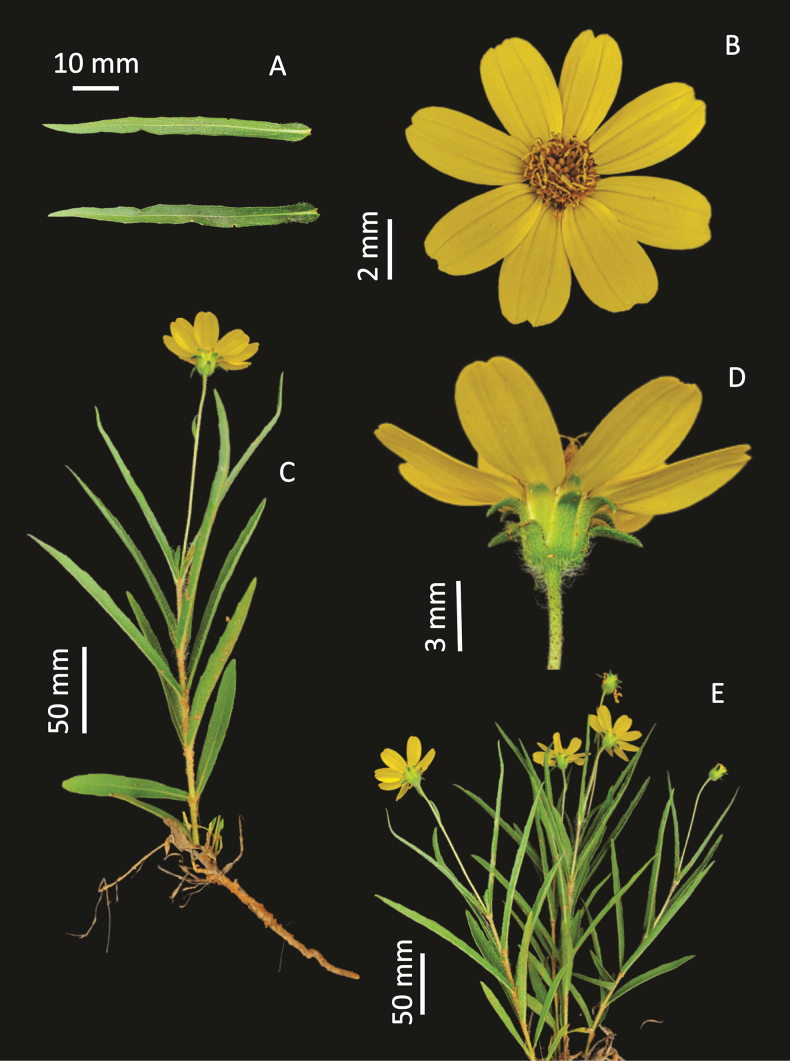
*Wedeliafigueiredoana* sp. nov. **A** linear blade leaf **B** head (top view) **C** flowering branch **D** head and phyllaries (side view) **E** habit.

**Figure 3.**
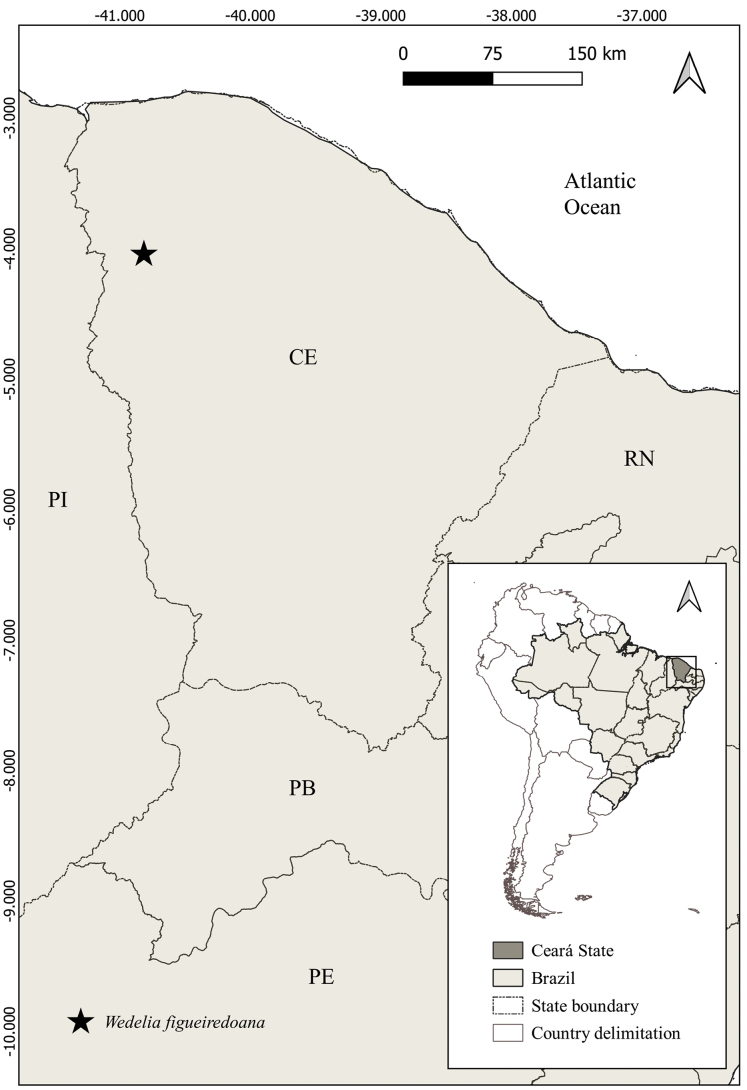
Geographic occurrence of *Wedeliafigueiredoana* sp. nov. in Brazil. CE: Ceará, PB: Paraíba, PE: Pernambuco, PI: Piauí, RN: Rio Grande do Norte.

**Figure 4.**
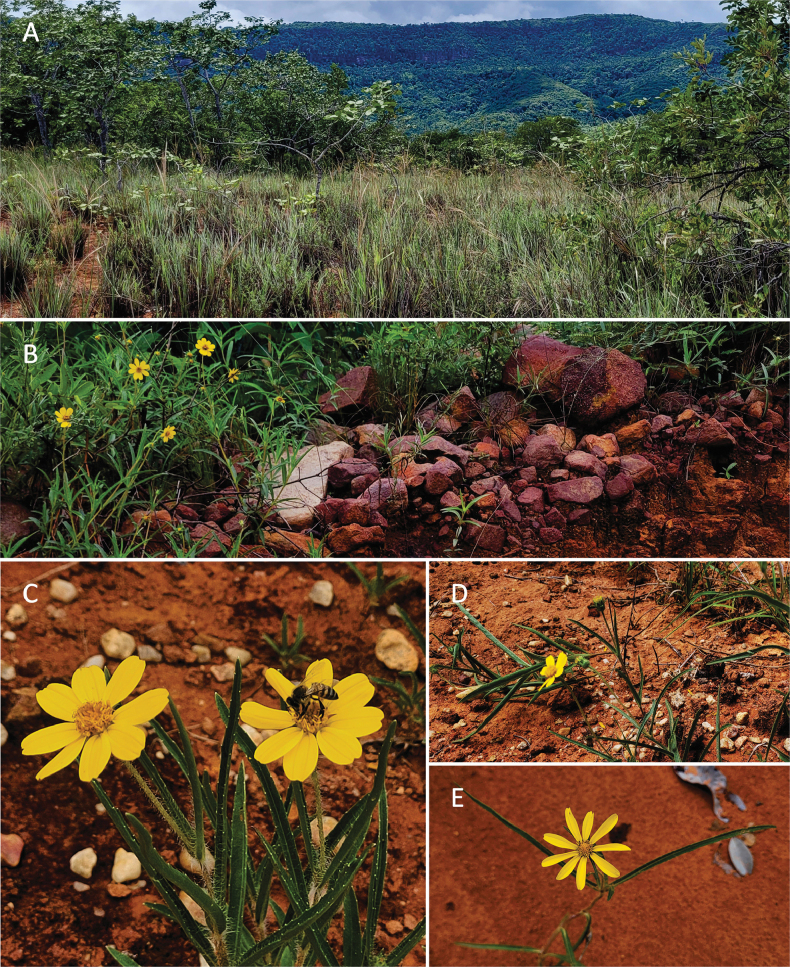
*Wedeliafigueiredoana* sp. nov. habitat **A** enclaves of Cerrado **B***Wedeliafigueiredoana* in the ferrugineous soil **C** floral visitor in the *W.figueiredoana***D** prostrate habit **E** solitary herbaceous individual of *W.figueiredoana*.

**Figure 5.**
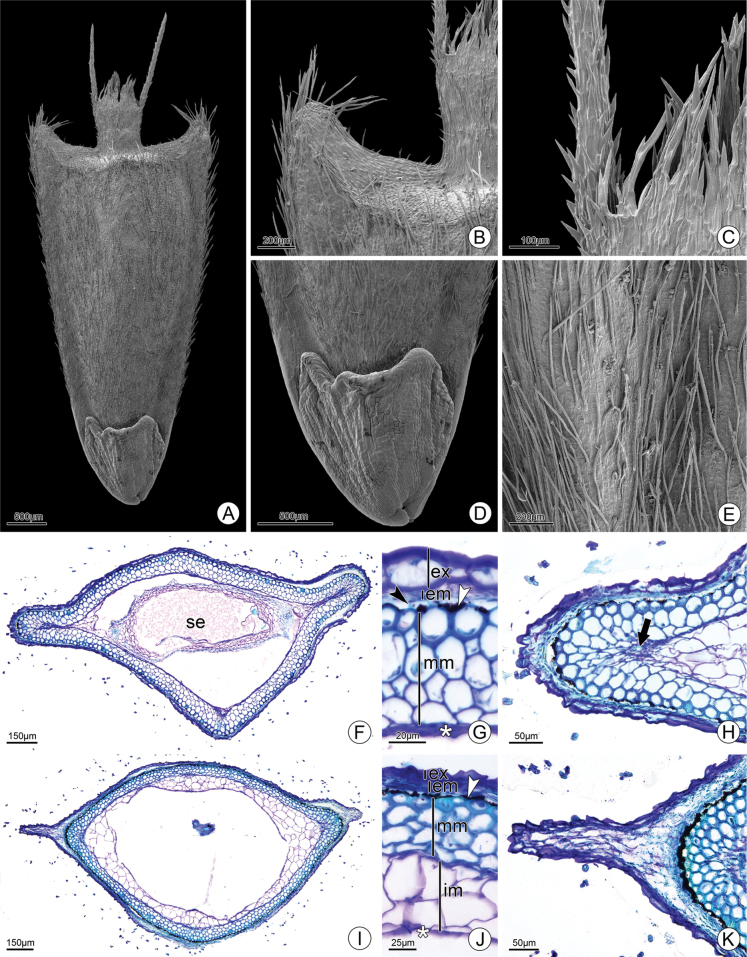
Cypsela of *Wedeliafigueiredoana* sp. nov. in scanning electron microscope (SEM) (**A–E**) and light microscopy (**F–K**). **A** General view **B** apex detail **C** coroniform pappus detail, note the outer awn and inner bristles fused at the base **D** basal region detail, observe the presence of elaiosomes **E** indumentum detail highlighting the biserial tector trichomes **F–H** middle region **F** overview **G** pericarp detail, note the sclereids of the middle mesocarp and the presence of phytomelanin **H** lateral region detail, observe the vascular bundle immersed in the mesocarp **I–K** cypsela apex **I** general view **J** pericarp detail **K** lateral region detail. in: external mesocarp; ex. exocarp; im: inner mesocarp; mm: medium mesocarp; se: seed; arrow: vascular bundle; asterisk: crashed layer (part of the inner mesocarp and endocarp); black arrowhead: sclereid; white arrowhead: phytomelanin. Cypsela analyzed from L. S. Rodrigues 201 (HUFU).

Figs 1 , 2 , 3 , 4 , 5

##### Type.

Brazil • **Ceará**: Graça, área de Cerrado rupestre; 4°05'18.1"S, 40°43'25.4"W; 26 February 2023; *L.S. Rodrigues 201* (holotype: HUFU00082144!, isotypes: HUVA!, HCDAL!, RB!).

##### Diagnosis.

*Wedeliafigueiredoana* morphologically resembles *W.bonplandiana* by 0.25–1.05 cm leaf blade width (vs. 1.5–2.6 cm), linear to narrow oblong leaf blade (vs. elliptic to spatulate), 3-seriate involucre (vs. 2-seriate), and cypselae 3.9–4 mm long (vs. 6–7 mm).

##### Description.

Herbaceous habit, prostrate, 0.15–0.3 m tall. Stems cylindrical, strigose to sparsely hirsute or densely hirsute, castaneous, internodes 0.84–6.7 cm long. Leaves decussate, rarely reduced in the base, sessile; blades 2.25–10.3 × 0.25–1.05 cm, linear to narrow oblong, base attenuate, apex attenuate, often acute, venation hiphodromous, often acrodromous basal, margins entire, flat; abaxial surface hirsute to densely strigose, veins hirsute to densely strigose, eglandular, adaxial surface hirsute to densely strigose, eglandular; olivaceous, concolorous to slightly discolorous, chartaceous. Capitula solitaries, terminal, peduncle (1.85–) 5.3–13.2 cm long, sparsely hirsute to hirsute, often strigose to sparsely hirsute, eglandular. Capitula heterogamous, radiate; involucre campanulate, (5.3–) 6.7–9.5 mm × 6.7–12.7 mm. Phyllaries 3-seriate; blades lanceolate, margins entire, flat, eglandular surfaces; outermost series apex acute or attenuate, densely hirsute to hirsute or densely hirsute to densely strigose, first series blades 9.4–12 × 1.9–2.3 mm, margin ciliate or not ciliate, olivaceous, foliaceous; second series blades 7.6–10.7 × 2.2–3.1 mm, margin ciliate or not ciliate, olivaceous or pale yellow to olivaceous, foliaceous or scarious with apex foliaceous; innermost series blades 5.5–7.6 × 1.6–2.6 mm, apex attenuate, sparsely hirsute to strigose, pale yellow to olivaceous, scarious with apex foliaceous or scarious. Receptacle flat, holopaleaceous, paleae 6.7–8.9 × 2–2.6 mm, narrow elliptic or narrow oblong, apex acute, concave or conduplicate, pale yellow to yellow. Ray florets 6–8, neutral, corolla ligulate, 10.3–14.3 mm long, tube 1.2–2.5 mm long, limb 9.1–12.3 × 6–6.3 mm, obovate or wide elliptic, apex 3-lobulate, 3–6 veins, tube pilose or glabrous, surface abaxial glabrous, surface adaxial glabrous, nerves sparsely pilose or glabrous, yellow. Disc florets 25–40, monoclinous, corolla tubular, 4.8–6.2 mm long, tube 1.3–1.9 mm long, lobes 0.9–1.1 mm long, glabrous, yellow; anthers 2.2–3 mm long, apical anther appendages ovate, black; style arms 1–1.2 mm long, linear, yellow. Cypselae 3.9–5.6 mm long, obovoid, flattened, densely sericeous, blackish, wings present throughout the pericarp, but apically pronounced, 0.04–0.4 mm long, yellow; pappus constrict at base, coroniform 0.5–0.6 mm long, 2-aristate, bitypic, monolength or bilength, 1.2–2.9 mm long, yellow.

##### Distribution and habitat.

*Wedeliafigueiredoana* is a microendemic species, which is known to occur exclusively in one municipality of Ceará state: Graça (Figs 3, 4). The new species grows in ferruginous open areas of Cerrado enclaves in elevations around 370–600 m a.s.l.. These enclaves also occur in the Ibiapaba plateau (Moura-Fé 2017), in the municipality of Graça, Ipu, Ipueiras, Pacujá, Pires Ferreira, and Reriutuba (municipalities from Ceará state). This new species occurs in testimonial hills and small inselbergs associated with the plateau (Moura-Fé 2017; Claudino-Sales et al. 2020), forming a Cerradão interspersed with open areas, rocky outcrops and ferruginous soils. This area is a transition zone between the Caatinga vegetation in low-elevation areas to the humid and subhumid forests in the high-elevation areas.

##### Conservation status.

The GeoCAT analysis (Bachman et al. 2011) considers that each collection point has 4.0 km^2^ of Area of Occupancy, but the species occurs in patches of open areas in the midst of closed vegetation, which greatly restricts its habitat. The Extent of Occurrence (Bachman et al. 2011) was not calculated because there were only two collection points available. Through satellite images and QGIS v.3.0 mensuration, we believe that the areas so far known for the species are not larger than 0.5 km^2^. According to the IUCN (2022) criteria, *W.figueiredoana* meets the following requirements for the Critically Endangered status: the criteria B1 (less than 100 km^2^ of extent of occurrence), B2 (less than 10 km^2^ of area of occupancy) and the condition A of criterion B (because it has one known location); criterion C (less than 250 mature individuals), conditions i and ii of C2 (about 50 individuals were seen, all mature individuals); criterion D (about 50 individuals were seen). Therefore, *Wedeliafigueiredoana* is proposed as Critically Endangered (B1; B2a; C, C2i, ii; D).

##### Phenology.

The specimens were collected with florets and fruits in February.

##### Etymology.

The epithet “*figueiredoana*” is in honor of Dr. Marlene Feliciano Figueiredo, born in 1963, a dedicated educator at Universidade Estadual Vale do Acaraú (UVA). Her dynamic engagement in teaching, research, and outreach, notably the Pensando Verde project, has left an indelible mark. Noteworthily, she was pivotal in founding and curating the Herbário Francisco José de Abreu Matos–HUVA (1998–2004), a vital botanical resource in Northwest Ceará. In botanical research, Figueiredo specializes in phanerogam taxonomy, floristics, ethnobotany, and seed germination ecophysiology, fostering a deep understanding of Northwestern Ceará’s intricate flora through her mentorship.

### ﻿Anatomical and morphological cypsela studies

The cypsela of *Wedeliafigueiredoana* is flattened-obovoid (Fig. 5A) with lateral wings (Fig. 5A, B). The pappus is coroniform (Fig. 5A–C) with two aristae and several smaller bristles setose (Fig. 5C). The carpopodium is conspicuous and bilobed with elaiosomes on both sides (Fig. 5A, D). Biseriate trichomes are distributed throughout the cypsela (Fig. 5E). In cross section, the cypsela is rhombic (Fig. 5F, I). The exocarp is composed of juxtaposed and periclinally flattened cells. The mesocarp is divided into three regions: the outer one is formed by approximately two parenchymatic layers, the median one has approximately five layers of sclereids and the inner one has composed of parenchymatic cells consumed in the median region of the cypsela and maintained in the apical region. (Fig. 5G, J). Four collateral bundles are immersed in the sclereids (Fig. 5H). The outermost layer of the median mesocarp has anticlinal projections that are responsible for the schizogenous space filled with phytomelanin (Fig. 5G, H, J, K). The endocarp appears consumed by seed growth. The wing is composed of the entire pericarp with a projection of the median mesocarp at the apex (Fig. 5H, K).

## ﻿Discussion

The herbaceous habit, decussate leaves, monocephalous heads, overlapped phyllaries in the involucre, and the neutral ray florets resembles the *Wedeliafoliacea* group: *Wedeliafoliacea* (Sprengel) B.L. Turner, *W.montevidensis* (Sprengel) B.L.Turner, and *W.riedelii* (Baker) B.L.Turner. However, *W.figueiredoana* can be differentiated from this group by the 3-seriate involucre (vs. 2-seriate, rarely 3-seriate), second series of phyllaries hirsute (glabrous, rarely pilose), and pronounced apically winged cypselae (vs. wings absent).

*Wedeliafigueiredoana*, *W.bonplandiana* (Gardner) B.L.Turner, *Wedeliabrachylepis* Griseb., and *Wedeliarudis* (Baker) H.Rob. are the unique Brazilian *Wedelia* species with winged cypselae. *Wedeliafigueiredoana* and *W.bonplandiana* have neutral ray florets, while *W.brachylepis* and *W.rudis* have pistillate ray florets. *Wedeliafigueiredoana* and *W.bonplandiana* are the only two species from Brazil with a pronounced apically winged cypselae, they also share more characters as the herbaceous and prostrate habit, sessile leaves, both surfaces with strigose indumentum, solitaries capitula, and paleae about 7 mm long (Santos 2001). Nevertheless, *W.figueiredoana* can be individualized from *W.bonplandiana* by 0.25–1.05 cm leaf blade width (vs. 1.5–2.6 cm), linear to narrow oblong leaf blade (vs. elliptic to spatulate), 3-seriate involucre (vs. 2-seriate), and cypselae 3.9–4 mm long (vs. 6–7 mm).

Blake (1931) was the first author who described “auriculate” projections to the *Wedelia* cypselae for *W.penninervia* S.F.Blake. Strother (1991) described three taxa with the same projections: W.acapulcencisvar.hispida (Kunth) Strother, *W.greenmanii* B.L.Turner, and *W.strigosa* Hook. & Arn. The same terminology was applied by Pruski and Robinson (2018) to describe the cypselae of *Wedeliafilipes* Hemsl. Until then, these were the only known *Wedelia* species with such structures described. The theoretically same structure can be seen in the cypselae of *W.bonplandiana* (Gardner) B.L.Turner. In the first moment of the morphological descriptions, it was believed to be the same projections in the cypselae of *W.figueiredoana*.

Strother (1991) and Pruski and Robinson (2018) made the distinction between species with winged cypselae and cypselae with auriculate projections. Our anatomical results found internal tissues in the supposed projections so we will treat these projections as wings. Based on this, we hypothesize that the species aforementioned with such projections also have winged cypselae, but further studies will elucidate this. We propose that this specific type of wing, described here for *W.figueiredoana*, be treated as “apically pronounced wings” to differentiate them from the wings that are more developed and that can be easily seen along the cypselae commonly found in *Wedelia* species, such as the wings described by Strother (1991).

Elaiosomes are more common in seeds but can be found in fruits of Asteraceae, such as *Centaurea* (Roth 1977). For *Wedelia*, elaiosomes proved to be very important for new classification of this genus, proposed by Alves (2019). In this classification, *Wedelia* now includes the genera *Aspilia*, *Coronocarpus* Schumach. & Thonn., *Oyedaea* DC., *Steiractinia* S.F. Blake, *Zexmenia* La Llave, *Zyzyxia* Strother, and two species of *Elaphandra* Strother (Alves 2019). According to Alves (2019), clades within *Wedelia* that do not present elaiosomes are considered as a derived character state. The anticlinal projections present in the outermost layer of the median mesocarp of *W.figueiredoana* were also described for *Clibadium*, *Desmanthodium*, *Ichthyothere* (Stuessy and Liu 1983) and *Calea* (Marques et al. 2022).

### ﻿Key to the species of Brazilian winged cypselae *Wedelia* species

**Table:** 

1	Herbaceous habit, prostrate or decumbent, shorter than 50 cm tall; ray florets neutral	**2**
–	Shrubby habit, erect, longer than 50 cm tall, ray florets pistillate	**3**
2	Leaf blade width 0.25–1.05 cm, linear to narrow oblong; 3-seriate involucre	***W.figueiredoana***
–	Leaf blade width 1.5–2.6 cm, elliptic to spatulate; 2-seriate involucre	***W.bonplandiana***
3	Stems hirsute or pubescent; leaf base attenuate	***Wedeliarudis***
–	Stems strigose; leaf base rounded	***Wedeliabrachylepis***

## Supplementary Material

XML Treatment for Wedelia figueiredoana

## ﻿Additional information

### Author ORCIDs

Vinicius R. Bueno https://orcid.org/0000-0001-9834-1951

Leonardo S. Rodrigues https://orcid.org/0000-0001-7541-9429

Francisco Diego Sousa https://orcid.org/0000-0002-0053-1333

Izaías C. Souza https://orcid.org/0000-0001-9877-2364

Juliana Marzinek https://orcid.org/0000-0002-4482-0655

Danilo Marques https://orcid.org/0000-0003-2571-9874

### Data availability

All of the data that support the findings of this study are available in the main text.
